# Engineering plant stress responses to combat climate change

**DOI:** 10.1111/nph.70836

**Published:** 2025-12-12

**Authors:** Matthew A. Jones

**Affiliations:** ^1^ Plant Science Group, School of Molecular Biosciences University of Glasgow University Avenue Glasgow G12 8QQ UK

**Keywords:** circadian, phenotypic plasticity, photoreceptor, retrograde signalling, thermosensor

## Abstract

This article is a Commentary on Bowerman *et al*. (2026), **249**: 1219–1233.

Climate change has already begun to limit crop growth due to elevated temperatures and increased frequency of climate events, including drought and heatwaves. These environmental changes present a particular challenge for plants due to their inherent phenotypic plasticity that allows adaptation to prevailing conditions. Should plants maximise their growth to increase fecundity or invest in strategies that improve stress tolerance? The difficulty of this developmental decision reveals a trade‐off between growth and resilience that is most dramatically observed in experiments that constitutively activate stress‐responsive signalling networks. This results in plants that are typically hardy yet smaller than their unmodified brethren, making this an imperfect solution for crops (Zhang *et al*., 2020). A new paper by Bowerman *et al*., published in this issue of *New Phytologist* (Bowerman *et al*., 2026; pp. 1219–1233), demonstrates how alternative approaches that instead manipulate plants' responses to environmental signals can influence development to increase field‐grown yield.

*Manipulation of retrograde signalling by Bowerman* et al. *provides an example of how responses to environmental signals can be engineered without inducing constitutive stress signalling*.

## Translating fundamental science into the field

Laboratory experiments are controlled by design, with limited variables to aid interpretation and often apply large stresses to induce measurable responses (Lundberg *et al*., 2025). By contrast, field experiments and practical applications are inevitably multifactorial and perhaps milder in magnitude, with at least light and temperature changing on a continual basis (Prado *et al*., 2025). Given this complexity, it is perhaps unsurprising that transgenic lines created and tested in the laboratory typically have reduced benefits in field trials (Prado *et al*., 2025). Changes in approach will be needed to improve the pipeline from laboratory to field, including the careful consideration of agronomically relevant responses when designing experiments to identify, characterise, and engineer climate‐resistant crops (Prado *et al*., 2025).

## Engineering responses to environmental change

A combination of agronomic approaches and crop improvement will be required to maintain and increase crop yield despite the consequences of global warming (Prado *et al*., 2025). Croplands are already moving away from the equator to avoid elevated temperatures while minimising irrigation requirements (Beyer *et al*., 2022). These geographical movements come with the caveat that changes in seasonal day length and temperature have increased magnitude away from the equator, which could result in impaired flowering time and altered development (McClung, 2021). Domestication has already selected varietals with altered circadian rhythms, and the alignment of crop chronotype with latitude provides opportunities to refine flowering time as farmers revise their planting strategies (McClung, 2021; Steed *et al*., 2021).

**Fig. 1:**
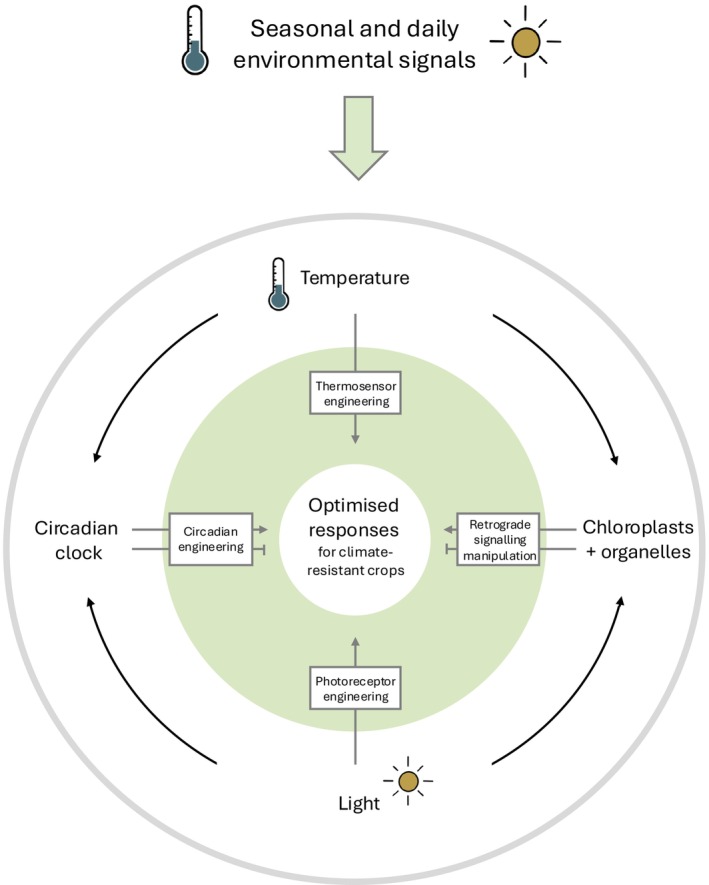
Engineering of plant responses to environmental stimuli provides opportunities to create climate‐resistant crops. Environmental signals, including temperature and light, are perceived by a combination of sensory proteins and the metabolic consequences of photosynthesis. Integration of these signals via a combination of anterograde and retrograde signals is modulated by the circadian system to facilitate short‐term and long‐term developmental responses. Understanding and engineering these responses will contribute to the development of climate change‐resistant crops.

An alternative target to combat climate change is to manipulate plants' responses to heat and drought stress (Fig. 1). Plants perceive environmental signals through a combination of receptor proteins and metabolic changes arising from photosynthesis and respiration (Jones, 2019; Queiroz *et al*., 2023). Temperature and light signalling are increasingly recognised as being interlinked, with the biological activity of many photoreceptors being temperature‐dependent (Kerbler & Wigge, 2023; Sharma *et al*., 2025). Alongside these dedicated environmental sensors, retrograde signals conveying the ‘operational status’ of the chloroplast (and mitochondria) have also been co‐opted through evolution to serve as environmental sensors. Photosynthates (or indeed toxic by‐products such as Reactive Oxygen Species) govern biochemical reactions elsewhere in the cell by providing chemical energy, cofactors, or other signalling molecules (Lee & Kim, 2024). The regulation of nuclear gene expression by these retrograde signals enables coordination of chloroplast function with other sensory pathways (Jones, 2019). Operational retrograde signalling has previously been thought to have a primary role in short‐term responses to stress. The question remains whether these short‐term retrograde signalling events are sufficient to induce longer‐term changes in growth and development (van Veen *et al*., 2025). In particular, it remains unclear how signalling pathways initiated during the initial stages of stress contribute to longer‐term developmental changes such as flowering time and yield.

## Priming retrograde signalling pathways to regulate developmental responses

Despite the opportunities to edit crop responses to environmental stress, we have comparatively few case studies that examine this strategy in practice. Bowerman *et al*. begin to address this hypothesis by examining the consequences of priming a retrograde signalling pathway to be more responsive to drought stress. SAL1 is an adenosine phosphatase that catabolises 3′‐PhosphoAdenosine 5′‐Phosphate (PAP) into adenosine monophosphate (Estavillo *et al*., 2011). Oxidative stress in the chloroplast (and/or mitochondria) inhibits SAL1 enzymatic activity, leading to accumulation of PAP in the cytosol with consequent pleiotropic effects upon transcription and RNA stability in Arabidopsis (Estavillo *et al*., 2011; Chan *et al*., 2016). Despite these pleiotropic effects, Arabidopsis mutants that constitutively accumulate PAP are more drought tolerant, with PAP accumulation inducing stomatal closure in parallel with abscisic acid signalling (Pornsiriwong *et al*., 2017). Bowerman *et al*. exploited genetic redundancy in wheat to create mutant lines with impaired SAL activity that accumulate greater amounts of PAP during stress while retaining low PAP levels in control conditions. Reduced SAL activity effectively lowers the threshold of PAP‐mediated retrograde signalling, promoting stomatal closure during periods of drought and increasing the yield of field‐grown wheat.

Manipulation of retrograde signalling by Bowerman *et al*. provides an example of how responses to environmental signals can be engineered without inducing constitutive stress signalling. There are also opportunities to modulate retrograde signalling by regulating photosynthesis, for example, by limiting nonphotochemical quenching (Zuo, 2025). Application of this approach to other environmental sensing pathways, for example, photoreceptor or thermosensor engineering, or modification of circadian clock activity, is an interesting avenue to create climate change‐resistant crops (Hart *et al*., 2019; Hu *et al*., 2020; Steed *et al*., 2021; Battle *et al*., 2024).

The toolkit of approaches to manipulate plant behaviour is now expanding, building on the evidence base that short‐term signalling responses can cumulatively control crop development. These examples illustrate how crucial fundamental research identifying stress‐responsive pathways can now be exploited to maintain crop production in the face of climate change.

## Disclaimer

The New Phytologist Foundation remains neutral with regard to jurisdictional claims in maps and in any institutional affiliations.
